# A Randomized, Double-Blind, Placebo-Controlled Study to Assess the Efficacy and Safety of a Nutritional Supplement (ImmuActive^TM^) for COVID-19 Patients

**DOI:** 10.1155/2021/8447545

**Published:** 2021-10-11

**Authors:** Muhammed Majeed, Kalyanam Nagabhushanam, Kalpesh Shah, Lakshmi Mundkur

**Affiliations:** ^1^Sami-Sabinsa Group Limited, 19/1, 19/2, I Main, II Phase, Peenya Industrial Area, Bangalore, Karnataka 560 058, India; ^2^Sabinsa Corporation, 20 Lake Drive, East Windsor, Piscataway, NJ 08520, USA

## Abstract

**Background:**

SARS-CoV-2 has emerged as a global threat due to its infectivity and rapid transmission. We evaluated the safety and efficacy of herbal and mineral formulation (ImmuActive) as an adjunct therapy in COVID-19 patients.

**Methods:**

A randomized, double-blind, placebo-controlled study was conducted in 100 COVID-19 patients in three centers in Southern India, and 92 subjects completed the study. Subjects were followed up until they were discharged from the hospital or for a maximum of 28 days, whichever was earlier. The primary outcome parameters were the mean change and time required to change the ordinal scale of disease severity by one unit. The secondary outcomes were the time required to turn RT-PCR negative or get discharged from the hospital, change in modified Jackson's Symptom Severity score, and COVID-19 quality of life questionnaire.

**Results:**

The ordinal scale at the end of the study was significantly lower in COVID-19 patients supplemented with ImmuActive (0.57) than placebo (1.0), with a *p* value of 0.0043. The ordinal scale decreased by one unit within 2.35 days in ImmuActive-supplemented patients, while it took 3.36 days in placebo-supplemented patients. Days of hospitalization and time required to turn RT-PCR negative were comparatively lower in the ImmuActive arm than the placebo arm. Change in modified Jackson's Symptom Severity Score and COVID-19 QOL were significant from screening to the end of the study in both ImmuActive and placebo arms. There were no adverse events observed during the study period.

**Conclusion:**

The study results suggest that ImmuActive could be a beneficial and safe adjunct treatment for effectively managing COVID-19 infection symptoms.

## 1. Introduction

Severe acute respiratory syndrome coronavirus 2 (SARS-CoV-2) infection or the coronavirus disease 2019 (COVID-19) pandemic has devastated the health and economy of the world since its first outbreak in late 2019 at Wuhan City, China [1].

Patients with COVID-19 generally develop mild symptoms of cold, cough, fever, and body ache. Individuals with comorbidities such as cardiac diseases, type 2 diabetes, obesity, and chronic kidney disease and the elderly tend to experience severe symptoms and a higher risk of severe illness [2]. Loss of smell and taste may be present as early symptoms. Persistent chest pain, blue lips, severe respiratory syndrome, kidney failure, and even death are observed [3, 4]. However, 80% of cases are estimated to be mild and do not progress to severe disease stages [5]. Although many existing and repurposed drugs have shown some potential in treating COVID-19, patients are treated symptomatically as no approved medication is available [6]. Despite at least four different approved vaccines, the COVID-19 infection continues with a second and third wave in several countries in different parts of the world [7, 8].

Complementary and alternative medicine (CAM) is generally used in conjunction with conventional medicine to decrease symptoms' severity and promote general well-being [9]. It is integrated with mainstream healthcare, especially for managing chronic diseases [10] like pain, osteoarthritis, diabetes, hyperlipidemia, and metabolic diseases [11–16]. In the absence of a specific cure for COVID-19, CAM is explored to reduce morbidity and complications during the infection [17]. The immune status of patients plays an essential role in the progression of COVID-19 infection. Herbal formulation with an immunomodulatory effect could potentially have a prophylactic effect and may even help as a therapeutic agent for COVID-19-infected patients [18]. The recent emergence of variant strains further accentuates the need for balanced immunity. Supportive or adjunctive therapies can help hasten recovery and reduce severe morbidity and mortality in COVID-19 patients. Chinese herbal medicines have played an essential role in tackling COVID-19 in China [19]. A recent systematic meta-analysis revealed that the combination of Chinese herbal medicine and conventional therapy could improve the clinical cure rate and reduce the rate of conversion to severe cases [20]. Since then, several herbal extracts and formulations are reported to be effective against SARS-CoV-2 infection [21]. The present study explored the effect of herbal and mineral formulation with known antiviral activities and nutritional effects on the immune system as an adjunct therapy in COVID-19 patients.

Curcumin and its analogs from *Curcuma longa* have shown potent antiviral activity through several pathways. Curcumin may modulate several molecular targets, including the ACE receptor and spike protein involved in the attachment and internalization of SARS-CoV-2 in different cells. Curcumin shows an inhibitory effect on SARS-CoV-2 replication in vitro in Vero cells [22, 23]. Numerous studies suggest that the curcuminoids inhibit proinflammatory cytokines such as IL-1, IL-6, IL-8, and TNF-*α*, prevent acute respiratory distress syndrome (ARDS), and repair the COVID-19-induced cellular damage [24, 25]. In a recent study, supplementation with curcumin and piperine could ensure early symptomatic recovery from infection.

*Andrographis paniculata* is traditionally used to support and optimize immune function, maintain healthy respiratory function, and ease seasonal allergies [26, 27]. The primary active molecule, andrographolides, is an inhibitor of the “Furin” protease in the lung, preventing spike protein activation and viral entry [28]. Andrographolides have also been shown to have antiviral activity by inhibiting viral entry, viral protein synthesis, viral replication, and viral protease activity in various viral diseases [29]. Recent in silico studies have predicted that andrographolides could inhibit the main protease activities of SARS-CoV-2 with the IC_**50**_ of 15 *μ*M [30, 31]. Resveratrol is an antioxidant that stimulates the immune system and downregulates the release of proinflammatory cytokines. It has shown a potential effect on SARS-CoV-2 by modifying the main pathways like the renin-angiotensin system (RAS) and expression of ACE2 [32]. In an in vitro study, resveratrol inhibited replication of SARS-CoV-2 in cultured Vero cells [33].

Selenium (Se) is an essential trace mineral for defense against viral infections, protection against oxidative stress, and protein folding [34]. The selenoproteins, glutathione peroxidases, and thioredoxin reductase are critical for antiviral defense as they maintain redox signaling and reduce oxidative stress. Selenium deficiency results in viral genome mutations to highly virulent forms and is associated with increased susceptibility and pathogenicity of viral infections [35, 36]. In a recent exploratory study, we observed reduced selenium levels in COVID-19 patients, thus supporting the rationale for this micronutrient's inclusion.

Zinc is an essential metal that can enhance both innate and humoral antiviral immunity. Zinc modulates the immune system by regulating T cells, natural killer cell activity, macrophage and neutrophil function, and T-cell-dependent antibody production [37]. Zinc also improves mucociliary clearance, reduces inflammation risk, and reduces lung damage and secondary infections [38–40]. Although piperine from *Piper nigrum* may have a viral inhibitory effect in COVID-19 patients, it is generally used as a bioavailability enhancer for natural products. Piperine was shown to have a binding affinity toward the spike glycoprotein of SARS-CoV-2 and its cellular receptor ACE2 [41].

The formulation containing curcuminoids, andrographolides, resveratrol, selenium, zinc, and piperine (ImmuActive^TM^) was evaluated along with the standard treatment of care in COVID-19 patients.

## 2. Materials and Methods

### 2.1. Design and Ethics

The study was conducted as a randomized, double-blind, placebo-controlled, multicenter, two-arm, prospective design. The efficacy and safety of ImmuActive^TM^ were assessed as an adjunct therapy for COVID-19 patients up to a maximum of 28 days or discharge from hospital/COVID-19 care center or transfer to ICU, whichever was earlier in comparison to placebo.

Adult (18–50 years) male and female COVID-19-positive patients with or without comorbid conditions such as diabetes and hypertension with BMI ≤ 35 kg/m^2^ were included in the study. The subjects who tested positive for COVID-19 by RT-PCR were enrolled within 48 hours. The enrolled patients had an ordinal scale score, less than or equal to 3, with the requirement of hospitalization or admission to the isolation ward, but stable with peripheral capillary oxygen saturation >94% on room air as described in the Ministry of Ayurveda, Yoga and Naturopathy, Unani, Siddha and Homoeopathy (AYUSH), Govt. of India, guidelines for designing COVID-19 clinical studies.

Asymptomatic COVID-19-positive patients with an ordinal scale of >3, under parenteral nutrition or tube feeding, or admitted to isolation ward or hospitalized for >48 hours of confirmed COVID-19-positive test were excluded from the study. Patients on ventilator support, with uncontrolled and unstable comorbidities, having a history of chronic lung disease, active malignancy, chronic kidney disease, and chronic liver disease, those who are immunocompromised, or those on immunosuppressants, allergic to investigational products, participating in another clinical study including macro/micro/any other forms of dietary supplements/multivitamins or oral nutrition supplements as well as pregnant and lactating females were excluded from the study.

Written informed consent was taken from all the subjects before enrollment in the study. The trial was conducted following the Declaration of Helsinki, the International Conference on Harmonization Guidelines for Good Clinical Practice, and applicable local regulations. The study included three centers, Prakriya Hospital (Bangalore), People Tree Hospital (Bangalore), and Apollo Hospital (Chennai), after approval from their respective Institutional Ethics Committee. The trial was registered prospectively on the Clinical Trial Registry of India (CTRI) with the registration number CTRI/2020/09/027841. The standard care of treatment was administered as per hospital treatment protocol and the respective state government's recommendations for managing COVID-19 patients. The treatment included antiviral (remdesivir and favipiravir), paracetamol, antibiotics (azithromycin/doxycycline), and multivitamins.

ImmuActive^TM^ 500 mg capsule containing curcuminoids (100 mg), andrographolides (50 mg), resveratrol (50 mg), zinc (10 mg), selenium (40 mcg), and piperine (3 mg) or placebo capsule (microcrystalline cellulose 500 mg) was administered orally to subjects once daily after breakfast in the morning (Table 1). Efficacy was evaluated for 28 days or discharge from hospital/COVID-19 care center or transfer to ICU, whichever was earlier.

The ordinal clinical severity scale was assessed every day until the negative RT-PCR test/discharge from the hospital or admission to ICU or a maximum of 28 days in both the intervention and control arm [42]. The scale has five different patient statuses: uninfected, ambulatory, hospitalized with mild disease, hospitalized with severe disease, and death (Table 2). The score was recorded as 0 when no clinical or virological evidence of infection was observed. Hospitalized with mild disease was further characterized as no limitation of activities (1), limitation of activities (2), and no oxygen therapy (3). The third scale of severe infection was characterized as oxygen by mask or nasal prongs (4), noninvasive ventilation or high-flow oxygen (5), intubation and mechanical ventilation (6), and ventilation + additional organ support-pressors, RRT, and ECMO (7). Death was given a score of 8. In the present study, subjects with less than or equal to a score of three were included. The mean change in ordinal scale from baseline to final visit was compared between ImmuActive and placebo. Further, the mean time required to reduce the scale by one unit was compared between the two groups.

Modified Jackson Symptom Severity Score was evaluated using a subjective self-reporting questionnaire for eight symptoms, which included sneezing, nasal discharge, nasal congestion, sore throat, malaise, fever, cough, and headache. Subjects rated each of their symptoms as follows: absent (0), mild (1), moderate (2), or severe (3). Subjects were assessed on randomization and the RT-PCR testing days until a negative RT-PCR result was established.

COVID-19 QOL questionnaires included six questions regarding the quality of the life of an individual. The QOL was recorded on the day of randomization, on day 6, on days when the RT-PCR test was conducted, and on the day of discharge/admission to ICU, whichever was earlier. Subjects were evaluated for their overall quality of life, mental health, physical health, and personal safety.

The mean number of days required to turn RT-PCR negative and the number of days of hospitalization were compared between the intervention and placebo arm.

The safety of the subjects through the incidence of adverse events was evaluated throughout the study period.

### 2.2. Statistical Analysis

The sample size was derived using population size, confidence level, and marginal error. The sample size was calculated to be 80 based on a confidence level of 95% and a marginal error of 5%. Allowing for a 25% dropout rate, the required sample size for recruitment was 100 in 1 : 1 ratio between two study groups (i.e., 50 per treatment group).

The continuous variables are presented as descriptive statistics of *n*, mean, standard deviation, and minimum and maximum values, whereas the categorical variables are presented as frequencies and percentages. A two-tailed independent samples *t*-test was performed to compare the treatment groups (ImmuActive^TM^ and placebo) to present the efficacy endpoint data. A two-tailed paired *t*-test was performed to compare the baseline data with the end of the study data for individual treatment groups. The efficacy parameters, namely, ordinal scale, modified Jackson's Symptom Score, and COVID-19 quality of life questionnaire, were compared between active and placebo arms. A chi-square test was conducted for categorical variables.

The differences in results obtained for the individual treatment groups between baseline and end of the study are presented as mean change from baseline and the *p* value derived through a two-tailed paired *t*-test. The two treatment groups were also compared in terms of the number of days taken for a change in disease severity on an ordinal scale by one, and the *p* value was derived through a two-tailed independent samples *t*-test.

A descriptive comparison of efficacy endpoints like the number of days of hospitalization and the number of days to negative RT-PCR reports is presented. A *p* value of <0.05 was considered statistically significant. Two decimal places are retained for all values. All the statistical analyses in the study were conducted using the STATA software version 16.0.

## 3. Results

### 3.1. Study Population

**Figure 1:**
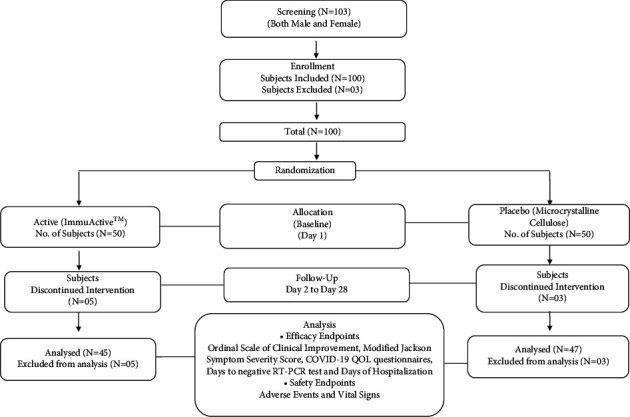
Consort diagram.

A total of 103 subjects were screened, and 100 (71 males and 29 females) were enrolled and randomized to *N* = 50 in the placebo and active arms. Eight subjects withdrew from the study, and 92 subjects (47 in placebo and 45 in ImmuActive arm) completed the study (Figure 1).

The mean age was 38.16 years among all subjects, 39.04 years in the active group, and 37.28 years in the placebo group at baseline. Patients' demographics were comparable between the groups. Vital signs were measured as a part of safety analysis, and no abnormal or out-of-range values were observed. The temperature ranged from 96.5 to 102.2°F, and SpO_2_ ranged from 95 to 99%. The detailed demographics and vital signs are presented in Table 3.

### 3.2. Primary Clinical Outcomes

#### 3.2.1. Change in Ordinal Scale

**Figure 2:**
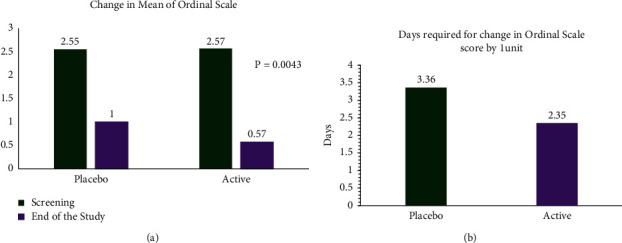
Change in ordinal scale. (a) The ordinal scale of disease severity was compared between placebo and ImmuActive groups from screening to the end of study. (b) Days required to reduce the disease severity by one unit.

The ordinal scale of disease severity significantly decreased from screening to the end of the study in the ImmuActive (2.57 to 0.57, *N* = 45) group compared to the placebo group (2.55 to 1.0, *N* = 47) with a *p* value of 0.0043 (Figure 2(a)). ImmuActive was found to have a significantly better (*p*=0.033) therapeutic response compared to placebo for the mean duration (days) required to reduce disease severity on the ordinal scale by 1 unit. Among the subjects receiving ImmuActive treatment, the mean duration was 2.35 days, whereas, in subjects receiving placebo, the mean duration was 3.36 days (Figure 2(b)).

### 3.3. Secondary Clinical Outcomes

#### 3.3.1. Clinical Evidence of Infection

At the end of the study, 19 patients (42.2%) in the ImmuActive group had no clinical or virological evidence of infection, and the ordinal score was recorded as 0, compared to 11 (23.4%) in the placebo group (*p*=0.03), which was statistically significant (Table 4).

#### 3.3.2. Days of Hospitalization

Length of hospital stay was marginally lower in the ImmuActive group (7.41 ± 1.79, median 6.5) than the placebo group (7.74 ± 2.35, median 7), and the difference was not statistically significant. The maximum number of days of hospitalization was ten days (median 6.5 (5–10)) in ImmuActive group and 15 days (median 7 (6–15)) in the placebo group. ImmuActive as an adjunct therapy reduced the maximum number of days in hospital by five days. Similarly, the number of days in hospital reduced in 10% population compared to the placebo group (Table 4).

#### 3.3.3. The Number of Days to Negative RT-PCR Report

The mean number of days to negative RT-PCR in the ImmuActive arm was 7.43 ± 2.11 and 7.89 ± 3.68 in the placebo arm. In the ImmuActive arm, 29 patients turned RT-PCR negative within six days, and the rest 16 turned negative by 14 days. In contrast, 27 patients in placebo turned negative in 6 days and 18 patients within 14 days, and two of them took more than 14 days to turn negative. The maximum number of days required to turn RT-PCR negative was 24 days in the placebo arm compared to 14 days in the active arm. Two patients from the placebo arm took more than 14 days to turn negative in the RT-PCR test (Table 4).

#### 3.3.4. Modified Jackson's Symptom Severity Score

Modified Jackson's Symptom Severity Score significantly decreased at the end of the study in both ImmuActive (4.98 ± 2.18 to 1.36 ± 1.71; *p* < 0.0001) and placebo groups (5.11 ± 2.29 to 1.70 ± 1.68; *p* < 0.0001) in comparison with the screening visit. However, the mean change in score was not statistically significant when compared between ImmuActive and placebo (Table 5).

#### 3.3.5. COVID-19 Quality of Life Questionnaire

A significant change in the mean of COVID-19 quality of life questionnaire was observed at the end in both ImmuActive (9.51 ± 3.25 to 6.82 ± 1.79; *p* < 0.0001) and placebo (9.06 ± 2.98 to 6.83 ± 1.30; *p* < 0.0001) groups in comparison with their screening visit (Table 4). Mean change in the COVID-19 quality of life questionnaire was similar in both the ImmuActive (−2.69) and placebo arms (−2.24) at the end visit (Table 6). The number of patients requiring antiviral therapy was comparatively higher in the placebo group.

### 3.4. Safety Analysis

A total of 100 subjects were enrolled, and 92 subjects completed the study; out of the 100 subjects, none reported adverse events in both ImmuActive and placebo groups. Eight subjects withdrew from the study for personal reasons.

## 4. Discussion

The present study shows that the herbal formulation ImmuActive may be beneficial as an adjunct therapy in COVID-19 patients to reduce the severity of the disease as assessed by the ordinal scale. The severity scale was significantly lower in treated subjects at the end of the study, and the time taken for a reduction in ordinal scale by one unit was significantly lower in ImmuActive supplemented patients. Further, 42.2% of the patients reported an ordinal scale of 0, suggesting a clinical and virological absence of infection in the ImmuActive arm compared to 23.4% in placebo. It is noteworthy that the median hospital stay required was five days more in the placebo than ImmuActive (10 versus 15). The burden of the pandemic in terms of resources is enormous for the patient and the healthcare system which is strained for funds and supplies. Under these circumstances, five days of lower hospital stay may offer considerable relief to the patients and the public health management. The hospital stays and time to turn viral negative were also lower in treated subjects compared to placebo.

ImmuActive is a formulation of active herbal extracts and micronutrients comprising curcuminoids, andrographolides, resveratrol, zinc monomethionine, L-selenomethionine, and piperine which are known to be helpful in viral diseases and have a positive impact on the immune system. Curcuminoids are proven to be anti-inflammatory agents with several clinical benefits. Curcumin was recently shown to bind to the main protease of severe acute respiratory syndrome coronavirus 2 (SARS-CoV-2) to inhibit viral replication [22]. It also prevents acute respiratory distress syndrome (ARDS) by inhibiting the cytokine storm, often seen in the terminal stages of the viral infection [43]. Supplementation with curcumin and piperine was beneficial in reducing the viral symptoms and maintaining oxygen saturation in COVID-19 patients in a clinical study. A systematic meta-analysis of randomized clinical trials using *A. paniculata* extract for acute respiratory tract infections revealed a shortened cough, sore throat, and time to resolution compared to usual care [44]. In another systematic analysis of seven double-blind controlled trials, *A. paniculata* was superior to placebo in alleviating uncomplicated upper respiratory tract infection [26]. Resveratrol is a stilbene with inhibitory activity against the replication of several pathogenic viruses [45]. Selenium supplementation enhanced the lymphocyte antioxidant activities and augmented host immune responses to infection in adult human subjects with relatively low serum selenium levels. Further, it improved natural killer cell activity and increased the percentages of activated T cells and antibody responses to vaccines [46]. In a recent exploratory study, we observed low selenium levels in COVID-19 patients [47]. Zinc is another essential trace element required to develop and activate T-lymphocytes, leading cells to defense against viral infections [48]. A significant number of COVID-19 patients were zinc deficient, with higher severity and complications than healthy individuals [49].

In the present study, the efficacy of ImmuActive was evaluated in COVID-19 patients with mild to moderate severity of the disease. Most of the patients were treated with antiviral drugs such as remdesivir and favipiravir as per the treatment plan prescribed by the local health authorities and hospital practice. Vitamin C and zinc supplements were also given to the patients as part of the treatment. Apart from mainstream therapy, few patients required mucodialaters and inhalers for symptomatic relief. We observed an increased number of patients in the placebo group (*N* = 24) who required either inhalers or mucolytic agents compared to the ImmuActive group (*N* = 16).

Despite the rigorous standard treatment modality, the significant improvement in the ordinal scale of disease severity suggests that supplementation with ImmuActive may benefit COVID-19 treatment. The formulation was not associated with any adverse effects, and no patients withdrew from the study due to any adverse effect of the formulation, suggesting the safety of ImmuActive in human subjects.

Our study has several limitations. Due to the state government's healthy agency's recommendations on the management of COVID-19 infection, we were unable to follow the patients for a long time to check the effect of ImmuActive on postrecovery morbidities associated with the infection. Although the study was conducted in three separate sites, our sample size seems small compared to the number of cases around the world.

The study was conducted in mild-moderate infection cases as this was the first clinical study using the formulation, and establishing safety was one of the primary criteria. The standard treatment in hospitals included potent antiviral drugs, which could have resulted in faster recovery of patients with mild symptoms.

## 5. Conclusions

In conclusion, the clinical study suggests that supplementation with ImmuActive with the standard treatment reduced the COVID-19 infection symptoms. The supplementation also significantly reduced the number of days spent in the hospital and the number of days required to turn viral negative by RT-PCR test. Significant improvement in ordinal scale suggests that the patients supplemented with ImmuActive moved from hospitalized stage to ambulatory, with no limitations on their activity compared to the placebo arm.

Further studies in larger populations and patients with different ranges of severity may be helpful to establish the benefit of ImmuActive as an adjunct therapy for COVID-19 infection.

## Figures and Tables

**Table 1:** Composition of ImmuActive^TM^.

S. no.	Ingredients	Label claim
1	C3 Reduct® ODN	100 mg
2	Andrographis paniculata extract >90%	50 mg
3	Resvenox® (resveratrol >95%)	50 mg
4	Zinc monomethionine (eq. elemental zinc)	10 mg
5	L-selenomethionine (eq. elemental selenium)	40 mcg
6	BioPerine®	3 mg

**Table 2:** Ordinal scale for clinical improvement.

	Patient state	Descriptor	Score

1	Uninfected	Uninfected; no viral RNA detected	0
2	Ambulatory	No limitation activities	1
		Limitation of activities	2
3	Hospitalized mild disease	Hospitalized; no oxygen therapy	3
		Oxygen by mask or nasal prongs	4
4	Hospitalized severe disease	Noninvasive ventilation or high-flow oxygen	5
		Intubation or mechanical ventilation	6
		Ventilation + additional organ support-pressors, RRT, and ECMO	7
5	Dead	Death	8

RNA: ribonucleic acid; RRT: renal replacement therapy; ECMO: extracorporeal membrane oxygenation.

**Table 3:** Screening/baseline demographic details and vital signs (mean ± SD).

Parameters	ImmuActive^TM^ (*N* = 50)	Placebo (*N* = 50)	*p* value
Age (years)	39.04 ± 7.70	37.28 ± 7.40	0.159 (NS)
Height (cm)	167.32 ± 6.40	166.18 ± 6.30	0.785 (NS)
Weight (kg)	67.96 ± 6.25	70.08 ± 7.56	0.304 (NS)
BMI (kg/m^2^)	24.32 ± 1.91	25.40 ± 2.14	0.064 (NS)
Systolic blood pressure (mmHg)	122.42 ± 7.54	123.30 ± 10.28	0.396 (NS)
Diastolic blood pressure (mmHg)	79.30 ± 7.72	80.82 ± 7.82	0.496 (NS)
Body temperature (°F)	98.43 ± 0.96	98.35 ± 0.72	0.678 (NS)
Pulse rate (beats/min)	90.56 ± 7.49	92.02 ± 7.32	0.474 (NS)
Oxygen saturation (SpO_2_) %	96.90 ± 0.99	97.06 ± 1.15	0.293 (NS)

NS, not significant.

**Table 4:** Secondary clinical outcomes.

Parameter	Active		Placebo		*p* value
	Number of patients	Percentage	Number of patients	Percentage	
*Ordinal scale*				
0	19	44.22	11	23.40	0.03
1	26	57.78	18	38.30	
2	0	0	16	34.04	
3	0	0	1	2.13	
4	0	0	1	2.13	

*Number of days in hospital*				
Less than 6	1	2.22	0		NS
6–10	44	97.78	42	89.36	
>10	0		5	10.64	

*No. of days of RT-PCR negative*				
6 days	29	64.44	27	57.45	NS
7–10	1	2.22	7	14.89	
10–14	15	33.33	11	23.40	
>14	0	0.00	2	4.26	

Patients with ordinal scale of “0”: no clinical evidence of infection. NS: not significant.

**Table 5:** Modified Jackson's Symptom Severity Score.

Parameter	Active (ImmuActive^TM^) group (*N* = 45)	Placebo (microcrystalline cellulose) group (*N* = 47)	*p* value
*Screening visit (visit 1)*			
Mean ± SD	4.98 ± 2.18	5.11 ± 2.29	0.7812(NS)
Minimum	2.00	1.00	
Maximum	10.00	11.00	
*End visit*			
Mean ± SD	1.36 ± 1.71	1.70 ± 1.68	0.3413(NS)
Minimum	0.00	0.00	
Maximum	8.00	5.00	
Mean change from baseline	−3.62	−3.41	
*p* value	<0.0001^*∗*^	<0.0001^*∗*^	

NS, not significant; ^*∗*^*p* < 0.05.

**Table 6:** COVID-19 quality of life questionnaire.

Parameter	Active (ImmuActive^TM^) group (*N* = 45)	Placebo (microcrystalline cellulose) group (*N* = 47)	*p* value
*Screening visit (visit 1)*		
Mean ± SD	9.51 ± 3.25	9.06 ± 2.98	0.4903 (NS)
Minimum	6.00	6.00	
Maximum	23.00	21.00	
*End visit*			
Mean ± SD	6.82 ± 1.79	6.83 ± 1.30	0.9757 (NS)
Minimum	6.00	6.00	
Maximum	17.00	11.00	
Mean change from baseline	−2.69	−2.24	
*p* value	<0.0001^*∗*^	<0.0001^*∗*^	

NS, not significant; ^*∗*^statistically significant.

## Data Availability

All data from the study are included in the manuscript.

## References

[1] Chakraborty C., Sharma A. R., Sharma G., Bhattacharya M., Lee S. S. (2020). SARS-CoV-2 causing pneumonia-associated respiratory disorder (COVID-19): diagnostic and proposed therapeutic options. *European Review for Medical and Pharmacological Sciences*.

[2] Center for Disease Control and Prevention Coronavirus disease 2019 (COVID-19)–symptoms. https://www.cdc.gov/coronavirus/2019-ncov/symptoms-testing/symptoms.html.

[3] Ali I. (2020). COVID-19: are we ready for the second wave?. *Disaster Medicine and Public Health Preparedness*.

[4] Jiang S., Xia S., Ying T., Lu L. (2020). A novel coronavirus (2019-nCoV) causing pneumonia-associated respiratory syndrome. *Cellular and Molecular Immunology*.

[5] Verity R., Okell L. C., Dorigatti I. (2020). Estimates of the severity of coronavirus disease 2019: a model-based analysis. *The Lancet Infectious Diseases*.

[6] Majedi S., Majedi S. (2020). Existing drugs as treatment options for COVID-19: a brief survey of some recent results. *Journal of Chemistry Letters*.

[7] WHO (2021). *WHO Lists Two Additional COVID-19 Vaccines for Emergency Use and COVAX Roll-Out*.

[8] Kim J. H., Marks F., Clemens J. D. (2021). Looking beyond COVID-19 vaccine phase 3 trials. *Nature Medicine*.

[9] Barnes P. M., Bloom B., Nahin R. L. (2008). Complementary and alternative medicine use among adults and children: United States, 2007. *Natural Health State Report*.

[10] Singer J., Adams J. (2014). Integrating complementary and alternative medicine into mainstream healthcare services: the perspectives of health service managers. *BMC Complementary and Alternative Medicine*.

[11] Askari A., Ravansalar S. A., Naghizadeh M. M. (2019). The efficacy of topical sesame oil in patients with knee osteoarthritis: a randomized double-blinded active-controlled non-inferiority clinical trial. *Complementary Therapies in Medicine*.

[12] Chang H. Y., Wallis M., Tiralongo E. (2011). Use of complementary and alternative medicine among People with type 2 diabetes in Taiwan: a cross-sectional survey. *Evidence-based Complementary and Alternative Medicine: eCAM*.

[13] Kopansky-Giles D., Vernon H., Boon H., Steiman I., Kelly M., Kachan N. (2010). Inclusion of a CAM therapy (chiropractic care) for the management of musculoskeletal pain in an integrative, inner city, hospital-based primary care setting. *Journal of Alternative Medicine Research*.

[14] Nayebi N., Esteghamati A., Meysamie A. (2019). The effects of a Melissa officinalis L. based product on metabolic parameters in patients with type 2 diabetes mellitus: a randomized double-blinded controlled clinical trial. *Journal of Complementary & Integrative Medicine*.

[15] Saydah S. H., Eberhardt M. S. (2006). Use of complementary and alternative medicine among adults with chronic diseases: United States 2002. *Journal of Alternative & Complementary Medicine*.

[16] Shagufta P., Asim K., Qamar K. (2020). Antihyperlipidemic effect of seeds of jamun (eugenia jambolana) in subjects of intermediate hyperglycemia: a pilot study. *Traditional and Integrative Medicine*.

[17] Seifert G., Jeitler M., Stange R. (2020). The relevance of complementary and integrative medicine in the COVID-19 pandemic: a qualitative review of the literature. *Frontiers of Medicine*.

[18] Chowdhury M. A., Hossain N., Kashem M. A., Shahid M. A., Alam A. (2020). Immune response in COVID-19: a review. *Journal of Infection and Public Health*.

[19] Hong-Zhi D., Xiao-Ying H., Yu-Huan M., Huang B.-S., Da-Hui L. (2020). Traditional Chinese Medicine: an effective treatment for 2019 novel coronavirus pneumonia (NCP). *Chinese Journal of Natural Medicines*.

[20] Du X., Shi L., Cao W., Zuo B., Zhou A. (2021). Add-on effect of Chinese herbal medicine in the treatment of mild to moderate COVID-19: a systematic review and meta-analysis. *PLoS One*.

[21] Benarba B., Pandiella A. (2020). Medicinal plants as sources of active molecules against COVID-19. *Frontiers in Pharmacology*.

[22] Zahedipour F., Hosseini S. A., Sathyapalan T. (2020). Potential effects of curcumin in the treatment of COVID‐19 infection. *Phytotherapy Research*.

[23] Maurya V. K., Kumar S., Prasad A. K., Bhatt M. L. B., Saxena S. K. (2020). Structure-based drug designing for potential antiviral activity of selected natural products from Ayurveda against SARS-CoV-2 spike glycoprotein and its cellular receptor. *Virus Disease*.

[24] Soni V. K., Mehta A., Ratre Y. K. (2020). Curcumin, a traditional spice component, can hold the promise against COVID-19?. *European Journal of Pharmacology*.

[25] Liu Z., Ying Y. (2020). The inhibitory effect of curcumin on virus-induced cytokine storm and its potential use in the associated severe pneumonia. *Frontiers in Cell and Developmental Biology*.

[26] Coon J. T., Ernst E. (2005). Andrographis paniculata in the treatment of upper respiratory tract infections: a systematic review of safety and efficacy. *Planta Medica*.

[27] Akbar S. (2011). Andrographis paniculata: a review of pharmacological activities and clinical effects. *Alternative Medicine Review: A Journal of Clinical Therapeutic*.

[28] Basak A., Cooper S., Roberge A. G., Banik U. K., Chrétien M., Seidah N. G. (1999). Inhibition of proprotein convertases-1, -7 and furin by diterpines of Andrographis paniculata and their succinoyl esters. *Biochemical Journal*.

[29] Gammoh N. Z., Rink L. (2017). Zinc in infection and inflammation. *Nutrients*.

[30] Enmozhi S. K., Raja K., Sebastine I., Joseph J. (2021). Andrographolide as a potential inhibitor of SARS-CoV-2 main protease: an in silico approach. *Journal of Biomolecular Structure and Dynamics*.

[31] Shi T.-H., Huang Y.-L., Chen C.-C. (2020). Andrographolide and its fluorescent derivative inhibit the main proteases of 2019-nCoV and SARS-CoV through covalent linkage. *Biochemical and Biophysical Research Communications*.

[32] Ramdani L. H., Bachari K. (2020). Potential therapeutic effects of Resveratrol against SARS-CoV-2. *Acta Virologica*.

[33] Yang M., Wei J., Huang T. (2021). Resveratrol inhibits the replication of severe acute respiratory syndrome coronavirus 2 (SARS‐CoV‐2) in cultured Vero cells. *Phytotherapy Research*.

[34] Rayman M. P. (2012). Selenium and human health. *The Lancet*.

[35] Guillin O., Vindry C., Ohlmann T., Chavatte L. (2019). Selenium, selenoproteins and viral infection. *Nutrients*.

[36] Kieliszek M., Lipinski B. (2020). Selenium supplementation in the prevention of coronavirus infections (COVID-19). *Medical Hypotheses*.

[37] Gammoh N., Rink L. (2017). Zinc in infection and inflammation. *Nutrients*.

[38] Pormohammad A., Monych N., Turner R. (2020). Zinc and SARS‑CoV‑2: a molecular modeling study of Zn interactions with RNA‑dependent RNA‑polymerase and 3C‑like proteinase enzymes. *International Journal of Molecular Medicine*.

[39] Sreeniwas Kumar A., Sinha N. (2020). Cardiovascular disease in India: a 360 degree overview. *Medical Journal Armed Forces India*.

[40] Wessels I., Rolles B., Rink L. (2020). The potential impact of zinc supplementation on COVID-19 pathogenesis. *Frontiers in Immunology*.

[41] Singh J., Malik D., Raina A. (2020). Computational investigation for identification of potential phytochemicals and antiviral drugs as potential inhibitors for RNA-dependent RNA polymerase of COVID-19. *Journal of Biomolecular Structure and Dynamics*.

[42] WHO (2021). *R&D Blueprint Novel Coronavirus COVID-19 Therapeutic Trial Synopsis*.

[43] Kedzierski L., Linossi E. M., Kolesnik T. B. (2014). Suppressor of cytokine signaling 4 (SOCS4) protects against severe cytokine storm and enhances viral clearance during influenza infection. *PLoS Pathogens*.

[44] Hu X.-Y., Wu R.-H., Logue M. (2017). Andrographis paniculata (Chuān Xīn Lián) for symptomatic relief of acute respiratory tract infections in adults and children: a systematic review and meta-analysis. *PLoS One*.

[45] Filardo S., Di Pietro M., Mastromarino P., Sessa R. (2020). Therapeutic potential of resveratrol against emerging respiratory viral infections. *Pharmacology & Therapeutics*.

[46] Hawkes W. C., Kelley D. S., Taylor P. C. (2001). The effects of dietary selenium on the immune system in healthy men. *Biological Trace Element Research*.

[47] Majeed M., Nagabhushanam K., Gowda S., Mundkur L. (2021). An exploratory study of selenium status in healthy individuals and in patients with COVID-19 in a south Indian population: the case for adequate selenium status. *Nutrition*.

[48] Wintergerst E. S., Maggini S., Hornig D. H. (2006). Immune-enhancing role of vitamin C and zinc and effect on clinical conditions. *Annals of Nutrition and Metabolism*.

[49] Jothimani D., Kailasam E., Danielraj S. (2020). COVID-19: poor outcomes in patients with Zinc deficiency. *International Journal of Infectious Diseases*.

